# Development of Cefotaxime Impregnated Chitosan as Nano-antibiotics: *De Novo* Strategy to Combat Biofilm Forming Multi-drug Resistant Pathogens

**DOI:** 10.3389/fmicb.2016.00330

**Published:** 2016-03-18

**Authors:** Bushra Jamil, Huma Habib, Shahid A. Abbasi, Ayesha Ihsan, Habib Nasir, Muhammad Imran

**Affiliations:** ^1^Microbiology and Public Health Group, Department of Biosciences, COMSATS Institute of Information TechnologyIslamabad, Pakistan; ^2^Center for Micro and Nano Devices, COMSATS Institute of Information TechnologyIslamabad, Pakistan; ^3^Department of Pathology, Al-Sayed HospitalRawalpindi, Pakistan; ^4^Industrial Biotechnology Division, National Institute of Biotechnology and Genetic EngineeringFaisalabad, Pakistan; ^5^School of Natural Sciences, National University of Sciences and TechnologyIslamabad, Pakistan

**Keywords:** biofilm, chitosan nano-carriers, cephalosporins, drug resistance, growth kinetics, zeta potential

## Abstract

Frequent incidents of antibiotic-resistant biofilm forming pathogens in community-associated and hospital-acquired infections have become a global concern owing to failure of conventional therapies. Nano-antibiotics (NABs) are *de novo* tools to overcome the multi-drug resistant mechanisms employed by the superbugs. Inhibition of biofilm formation is one of those strategies to curb multi drug resistance phenomenon. In the current study, the anti-biofilm and antibacterial potential of newly synthesized cefotaxime loaded chitosan based NABs have been investigated. Both bare and cefotaxime loaded NABs were prepared by ionotropic gelation method. They were found carrying positive zeta potential of more than +50 mV, indicating highly stable nano-dispersion. Moreover, microscopic studies revealed their size as less than 100 nm. NABs were tested against clinical isolates of multi drug resistant *Klebsiella pneumoniae, Pseudomonas aeruginosa, Escherichia coli, and* methicillin resistant *Staphylococcus aureus* and wherein they demonstrated broad-spectrum anti-biofilm and anti-pathogenic activity. Thus, *in vitro* synergistic action of cephalosporin drugs and chitosan polymer at nano-scale in contrast to free antibiotics can be an improved broad-spectrum strategy to thwart resistance mechanisms in both Gram-positive and Gram-negative resistant pathogens.

## Introduction

Though, it is a mutually agreeable fact that bacteria has no definite nervous system but it is also conceded that they evolve certain survival mechanisms that allow them to exist in any environment. Biofilm formation is one of such survival mechanisms. Most of the bacterial species prefer to live in a well-developed community (biofilm) rather than planktonic form (isolated individual cells; Dheilly et al., 2010; Sayem et al., 2011). Along with numerous other benefits, biofilms also provide the basic mechanism of resistance to antibiotics, antibodies, bacteriophages, disinfectants and other host defense systems (Dheilly et al., 2010) by constituting a multi-layered protection mechanisms (Stewart, 2002). Extracellular polymeric substance (EPS) covering the biofilms make them impermeable to many antibiotics. Likewise, in biofilms microbes exhibit slow growth rate which further makes them resistant to therapeutic agents. Furthermore, biofilm forming microbes develop adaptive stress responses which collectively evince another defense mechanism. The formation of persister cells are further addition to this menace and is the main cause of recurrent and chronic infections (Lewis, 2005). Biofilms also facilitate the horizontal or lateral gene transfer (Madsen et al., 2012) which is considered a significant feature in the evolutionary process of acquiring antibiotic resistance genes between species. Biofilms also provide a wide variety of genetic elements (plasmids) to be transferred among unrelated bacterial species including genes that promote biofilm formation and responsibles for resistance (Donlan, 2002).

Extracellular DNA (eDNA) which is formed by the autolysis of a microbial subpopulation also makes an integral part of microbial biofilms and plays a foremost role in the stability of the biofilms (Watnick and Kolter, 2000). These biofilms accumulate large quantity of antibiotic degrading enzymes too (Delcour, 2009). In addition, biofilm formation is associated with the virulence of pathogenic bacteria, and cells included within a biofilm are generally 1000 times more resistant. National Institutes of Health (NIH) has estimated that 70% of all microbial infections in the world are associated with biofilms (Perumal and Mahmud, 2013). It is, therefore, a major concern not only in health care systems but in certain other domains as well. It has also been a major source of contamination in the food industry, water supply pipes, medical implants, catheters and equipment etc (Dheilly et al., 2010; Perumal and Mahmud, 2013).

The effectiveness of many antimicrobial agents is currently decreasing owing to increasing prevalence of multidrug-resistant (MDR) pathogens (Fabbretti et al., 2011). The emergence of these MDR pathogens remains a serious challenge to medicine and healthcare systems (Carlet et al., 2012). One of the mechanisms for such resistance is the formation of biofilms. If antibiotics cannot traverse the biofilm they fail to eradicate other defense strategies. Therefore, it is important to search for alternative therapeutics to control biofilm-associated MDR infections (Perumal and Mahmud, 2013; Kirker et al., 2015).

The development of anti-biofilm and anti-MDR strategies is therefore a major area of interest and currently constitutes an important field of investigation. Albeit, various naturally occurring compounds (including plant extracts) have been investigated in this regard (Salta et al., 2013). However, nano-antimicrobials (NAMs) are offering more promising future to beat MDR phenomenon (Allaker and Memarzadeh, 2014; Jamil et al., 2015). NAMs can also overcome resistance caused by biofilm formation and can prevent biofilm’s further growth as well (Jamil et al., 2015). Recently, various NAMs have displayed their anti-biofilm potential to combat resistance mechanisms. Liposomes help thrashing biofilms by promoting adsorption at the outer surfaces of biofilms (Smith, 2005). Besides, silica nano-particles (NPs) act by releasing large amount of Nitric oxide (NO; Hetrick et al., 2009). Analogously, ZnO, TiO_2,_ MgF_2_ NPs and super paramagnetic iron oxide NPs (SPIONs) have also inhibited biofilm formation (Pelgrift and Friedman, 2013). The rationale behind successful eradication of biofilms lies in strong interaction between biofilms and NAMs. The majority of NAMs bear positive charge that attaches firmly to biofilm matrixes carrying negative charge (Hajipour et al., 2012). However, due to metallic NPs associated toxicity issues, nano-medicine research focus has been shifted toward bio-based NPs or nano-carrier systems (NCS). In general, NCS protects the drug from both endogenous and exogenous factors and provide a sustained release and more enhanced bioactivity.

Cefotaxime is a third generation broad spectrum cephalos porins for parenteral administration with a short half-life of 0.8–1.4 h, it is bactericidal and mainly used in the treatment of infections caused by Gram-positive and Gram-negative microorganisms. Very recent and frequent emergence of extended spectrum beta lactamases (ESBL) and metallo beta lactamases (MBL) are reducing the susceptibility of all cephalosporins including cefotaxime. Therefore, present investigation has been carried out in an attempt to enhance the bactericidal activity of this β-lactam antibiotic synergistically with antimicrobial bio polymer chitosan to render it more effective against biofilm producing MDR pathogens.

## Materials and Methods

Chitosan-medium molecular weight was purchased from Sigma–Aldrich (Product number 448877, 75–85% deacetylation, 200–800 cP viscosity of 1% w/v in 1% v/v acetic acid). Pentasodium triphosphate (TPP) and glacial acetic acid were also procured from Sigma–Aldrich. All antibiotic disks were obtained from Oxoid. Nutrient agar and nutrient broth were purchased from Oxoid. Cefotaxime sodium for injection (0.5 g) was procured from Sanoffi Aventis Pakistan Limited. Standard stock solution of cefotaxime sodium was prepared by dissolving 1 mg/mL in sterilized water.

### Culture Collection

The clinical isolates were obtained from Al-Sayed Hospital (Pvt) Ltd, Rawalpindi and stored at -80°C in nutrient broth containing 20% glycerol. Their identities were confirmed by biochemical test using API (analytical profile index) kits. For the purpose of this study, we had collected pathogenic bacteria which were capable of biofilm formation and were documented as causative agents for severe infections. Clinically resistant pathogens used in this study includes *Klebsiella pneumoniae* (KP), *Pseudomonas aeruginosa, Escherichia coli* (*E. coli*), *and* Methicillin resistant *Staphylococcus aureus* (MRSA). We could not get clinical isolates of *Listeria monocytogenes*, so *L. monocytogenes* ATCC 13932 had been employed for this study.

Inoculum was prepared by the method described in literature with slight modifications. All the bacterial strains were recovered on a fresh nutrient agar (Oxoid) plate 24 h prior to antimicrobial test. To prepare the inoculum, colonies from fresh agar were transferred into sterile Mueller Hinton (MH) liquid growth medium and incubated at 37°C overnight. The optical density was adjusted to 0.1 at 600 nm wavelength (Perumal and Mahmud, 2013).

### Resistance Spectra by Disk Diffusion Method

Selected microbes were subjected to antibiotic disks by standard Kirby Bauer method and their resistance patterns were studied and compared to CLSI guidelines. Following antibiotic disks were used including Ceftazidime (CAZ), Cefotaxime (CTX), Imipenem (IPM), Cefepime (FEP), Ceftriaxone (CRO), Aztreonam (ATM), Ampicillin (AMP), Vancomycin (VA), Augmentin (AMC), Colistin (CT), Cefoxitin (FOX), and Minocycline (MH). All selected bacterial cultures were marked as resistant or susceptible based upon their zones of inhibition.

### Quantification of Antibiotics by Nanophotometer

UV-Vis spectrophotometer (Nanophotometer Implen) was used for this purpose (El-Shaboury et al., 2007; Bushra et al., 2014). Volumetric dilutions of cefotaxime were prepared in triplicate from stock solution. AAAA_max_ was obtained after the wave scan for 200–900 nm range. Calibration curve was constructed with the average absorbance of three replicates. The trend line was obtained by linear regression with standard equation and R squared value.

### Chitosan Nanoparticles (CSNPs) Fabrication

Ionotropic gelation method was used to fabricate Chitosan Nanoparticles (CSNPs; Du et al., 2009; Jamil et al., 2016). Ionotropic gelation is based on the ability of poly electrolytes to cross link in the presence of counter-ions to form hydrogel beads also called as gelispheres (Patil et al., 2012). These gelispheres have the ability to control the release of drug. Cefotaxime solution was prepared in tripolyphosphate (TPP) and it was then added dropwise in chitosan (CS) solution with constant stirring. The TPP diffuses slowly into the CS solution forming a three dimensional lattice of ionically crosslinked moiety that has entrapped drug in it (Patil et al., 2012). It was then subjected to sonication for another 30 min by using Sonozap ultrasonic homogenizer 25 kHz. These NPs were then centrifuged at 12000 *g* for 10 min.

### Nanoparticles Characterizations

The detection and characterization of nanoparticles entail particular challenges. As particle size is a key criterion in nanotechnology, maintaining particle size both *in vitro* and *in vivo* is crucial. Stability of NPs is determined mainly by their size maintenance. Flocculation and aggregation due to van der Waals forces should be avoided as the behavior of NPs is remarkably different from the bulk material of the same matter. Bulk material does not change its physical properties as the size of matter changes, while the NCS display size dependent properties. As a general rule, extent of size reduction is directly proportional to the surface area. Increased surface area contributes toward greater activity.

### Scanning Electron Microscopy (SEM)

Scanning electron microscopy uses electrons instead of light to form an image and is one of the most widely used techniques for the characterization of nanostructures. Jeol JSM 6490A analytical scanning electron microscope was used for this purpose. Sample was prepared by placing a small drop of formulation on glass slide. Gold coating by Jeol Quick Auto Coater (JFC-1500) ion sputtering device was done for 6 s only. Analyses of both empty and drug loaded nanoparticles were carried out at the resolution of 20 kV and 3000–50000× magnification by the procedure already published (Jamil et al., 2016).

### Atomic Force Microscopy (AFM)

SEM gives a 2D image while AFM can provide a 3D topographic picture of prepared nano-systems. Agilent Pico Plus was used for this purpose. The AFM probe has a very sharp tip, often less than 100 Å diameter, at the end of a small cantilever beam. The probe is attached to a piezoelectric scanner tube. Inter-atomic forces between the probe tip and the sample surface cause the cantilever to deflect as the sample’s surface topography or other properties change. A laser light reflected from the back of the cantilever measures the deflection of the cantilever. Based on the type of application, different operation modes of AFM are used like the contact mode, semi contact mode and tapping mode (Suresh, 2015). However, for this study, all the images were taken in tapping mode at ambient conditions.

### Fourier Transform Infrared Spectroscopy (FTIR) Spectra

Fourier Transform Infrared Spectroscopy was done for qualitative analysis of prepared formulations. Infrared spectroscopy is associated with vibrational energy of atoms or group of atoms in a material. It gives peaks for each functional groups and also gives a clue about bonding and interactions. Perkin Elmer FTIR spectroscope was used to analyze dry sample after preparing their pallet with KBr (FTIR grade Merck) while liquids were analyzed directly. The spectral resolution was 4 cm^-1^, with 96 scans, and an aperture of 4 mm. The optimum beam incidence angle was 45° and all the spectra were acquired in a range between 4000 and 600 cm^-1^.

### Determination of Zeta Potential

Zeta potential was measured to get an indication about long term stability of nano-formulations. Zetasizer Nano ZS (Malvern Instruments, UK) using Doppler electrophoresis as the basic principle of operation was used in this study. The data collected were then imported to Excel and the means and standard deviations of the replicate measurements were calculated.

### Determination of Encapsulation Efficiency

The encapsulation efficiency (EE) of nanoparticles was calculated by the method earlier mentioned (Rotar et al., 2014). Drug loaded CSNPs were isolated from the free drug by centrifugation (12,000 *g* for 15 min, Eppendorf 5415D, Germany). Free drug in the supernatant was quantified by spectrophotometer at AAAA_max_ (298) obtained from wave scan. The concentration of drug in the supernatant was estimated by the equation obtained from standard curve. Experiments were performed in triplicate and the EE was calculated as follows:

E⁢E=(t⁢o⁢t⁢a⁢l⁢ d⁢r⁢u⁢g−u⁢n⁢ e⁢n⁢c⁢a⁢p⁢s⁢u⁢l⁢a⁢t⁢e⁢d⁢ d⁢r⁢u⁢g)÷t⁢o⁢t⁢a⁢l⁢  d⁢r⁢u⁢g×100

### Antimicrobial Potential of the Prepared Nano-antibiotics (NABs)

Antibacterial activity of blank and β-lactam antibiotic loaded CSNPs were evaluated according to standard liquid micro-dilution susceptibility assays. To this aim, selected microbes were grown in nutrient broth until the exponential growth phase at 37°C while shaking. Bacterial turbidity was compared to Mc-Farland solution. Ten microliters of the bacterial suspension were added to 9 mL broth and similar concentration of antibiotic and drug loaded CSNP were added in inoculated broth. Test tubes were incubated in shaker incubator at 37°C overnight. Optical densities values were taken after every 24 h for 144 h at 595 nm on Elisa Multi-plate reader (Jamil et al., 2016). Both positive and negative controls were added in this assay. Negative control contains only the nutrient broth without culture and served as control of any possible contamination while the positive control contains nutrient broth and inoculum to serve as a control of cell-viability.

At the end of experiment colony forming unit (CFU) assay was done to confirm the results. Serially diluted bacterial suspensions were plated on Nutrient agar surface and incubated for 48 h at 37°C. Results were expressed as log10 CFU/ml.

### Anti-Biofilm Potential of the Prepared Nano-antibiotics

To assess the anti-biofilm activity of cephalosporin loaded CSNPs against clinically important pathogens; MicroTiter Plate (MTP) assay was carried out using 96-well flat bottom polystyrene titer plates (Kirker et al., 2015).

Biofilms were formed on ELISA plate by the method of George with few modifications (George, 2011). Briefly, all selected pathogens were cultured on fresh Petri plate from stocks preserved at -80°C and from that pure media inoculum was prepared. Turbidity was compared to that of McFarland turbidity standard and 10 μL inoculum was added in test tube containing 9 mL of freshly sterilized nutrient broth. Nano-antibiotic and simple antibiotic solution prepared at same concentration were then added in each test tube. Approximately 200 μL of each sample was added in each well of 96 well ELISA dish. Each sample was prepared in triplicate and incubated at 37°C. After incubation, planktonic cells were removed by turning the plate upside down. It was then submerge gently in a small tub of water and water was removed. Washing was done thrice to remove all the unattached cells and also to lower background staining. Afterwards 200 μL of a 0.1% solution of crystal violet was added to each well of the microtiter plate. It was left in incubator for 10–15 min. Crystal violet solution was removed by tilting the plate and by rinsing with water 3–4 times. It was then blot dried.

To quantify the biofilms, 200 μL of 30% v/v acetic acid solution was added to each well to completely solubilize the crystal violet. The microtiter plate was incubated at room temperature for 10-15 min. The resultant solution was transferred to a new microtiter dish and absorbance reading was taken in a micro-plate reader at 595 nm (George, 2011; Perumal and Mahmud, 2013).

## Results

### Resistance Spectra by Disk Diffusion Method

**FIGURE 1:**
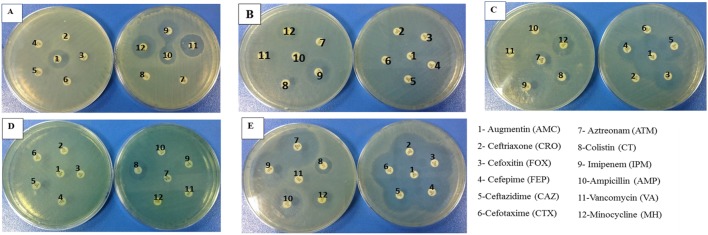
**Zones of Inhibitions formed on nutrient agar surface by different antibiotic disks against (A) Methicillin-resistant *Staphylococcus aureus* (MRSA), (B) *Klebsiella pneumoniae* (C) *Escherichia coli* displaying extended spectrum beta lactamases (ESBL) phenomenon exhibited by extension in zone of inhibition formed between augmentin and cephalosporins (D) *Pseudomonas aeruginosa* and (E) *Listeria monocytogenes*, after incubation at 37°C after 24 h**.

Resistance spectra of selected microbes were determined by standard disk diffusion method. Results were compared to CLSI guidelines to categorize organisms either as susceptible or resistant. Cefoxitin was used as an indicator of methicillin susceptibility disks and an inhibition zone diameter of ≤14 mm was reported as methicillin resistant. According to our results, *S. aureus* was resistant to cefoxitin (FOX) that is why we marked it as Methicillin Resistant *S. aureus* (**Figure 1**). It was also resistant to whole range of tested cephalosporins (**Table 1**). Likewise, *K. pneumoniae* was also resistant to all cephalosporins and augmentin as well, but was susceptible to Imipenem (IPM), Colistin (CT) and Minocycline (MH). *E. coli* was found to be ESBL positive as the inhibition zone expansion was observed with Augmentin (**Figure 1C**). In case of *P. aeruginosa*, it was susceptible to Colistin (CT) only otherwise it was observed to be highly resistant to all other tested antibiotics. So all the clinical pathogens were found to be highly resistant to the available therapeutic options. However, *L. monocytogenes* (ATCC 13932) was susceptible to all tested antibiotic disks except vancomycin.

**Table 1:** Zones of Inhibition formed by clinical isolates against different tested antibiotic disks.

Pathogens	AMC	CRO	FOX	FEP	CAZ	CTX	ATM	CT	IPM	AMP	VA	MH
	
	Zones of inhibition (mm)								
MRSA	18	–	–	–	–	–	–	–	17	15	20	20
*Klebsiella pneumoniae*	–	–	–	–	–	–	–	15	23	–	–	15
*Escherichia coli*	–	–	24^∗^	16^∗^	19^∗^	–	15	14	28	–	–	18
*Pseudomonas aeruginosa*	–	–	–	–	–	–	–	16	12	–	–	–
*Listeria monocytogenes* (ATCC 13932)	30	29	26	29	29	30	33	15	30	25	–	16

*Where Augmentin (AMC), Ceftriaxone (CRO), Cefoxitin (FOX) Cefepime (FEP), Ceftazidime (CAZ), Cefotaxime (CTX), Aztreonam (ATM), Colistin (CT), Imipenem (IPM), Ampicillin (AMP), Vancomycin (VA), and Minocycline (MH). ESBL phenomenon exhibited by increase in zone of inhibition. Whereas (–) means ‘no zone of inhibition*.

*^∗^indicates ESBL phenomenon marked by increase in zone of inhibition caused by AMC and cephalosporin discs*.

### Characterization of Nano-Particles

**FIGURE 2:**
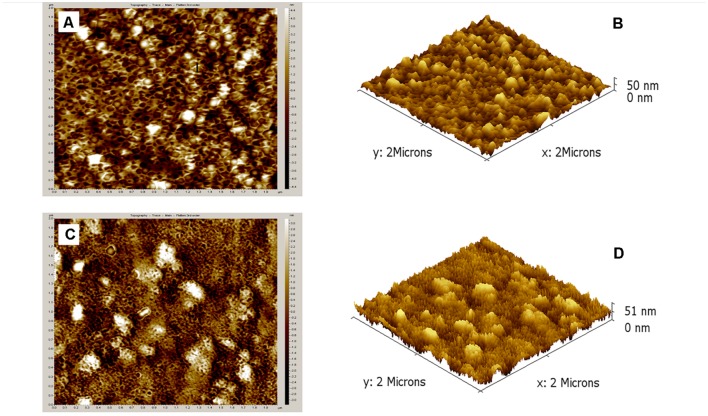
**Atomic force microscopic images of surface topography and 3-Dimensional (3D) structures of empty CSNPs (A,B) and cefotaxime loaded CSNPs (C,D) respectively.** A small drop of sample was dried on glass slide and all images were taken at room temperature without any sample treatment. Results obtained depict height of 50 and 51 nm.

**FIGURE 3:**
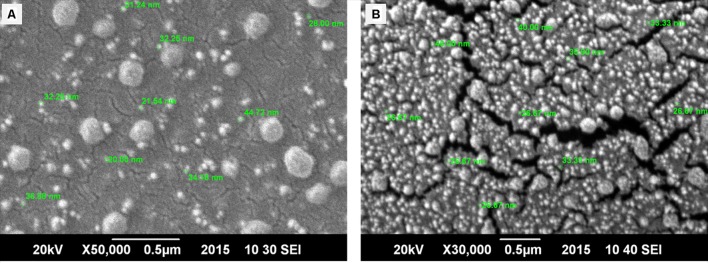
**Scanning electron microscopy (SEM) of bare (A) and drug loaded CSNPs (B).** SEM images were taken by placing a tiny droplet of sample on l × l cm glass slide and it was spreaded evenly. After gold sputtering, images were taken at ambient conditions. SEM images depicted spherical particles having diameter of less than 100 nm.

Ultramicroscopic characterization of nanoparticles was done to gather a firsthand information on particles morphology, size and distribution. AFM was performed to get surface topography and size distribution images of nano-formulations. 3D AFM images of both nano-systems revealed that both nano-formulations were homogeneously dispersed and a maximum height of 50 nm was observed for empty CSNPs and 51 nm for cefotaxime loaded CSNPs (**Figure 2**). Both empty and loaded nano-formulations were found to be quite uniform. However, cefotaxime loaded CSNPs were displaying more dense population. SEM results revealed that empty and cefotaxime loaded NPs were in the size range of less than 100 nm. Both AFM and SEM analysis demonstrated that there was more concentrated and more dense population of drug loaded NPs as compare to blank NPs (**Figure 3**).

### FTIR Spectra

**FIGURE 4:**
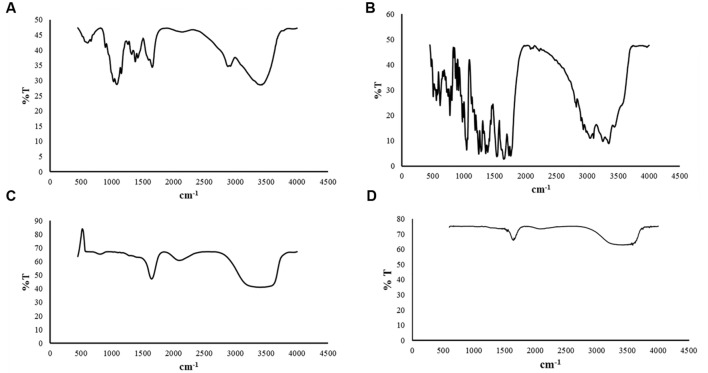
**Fourier Transform Infrared Spectroscopy spectra (A) Chitosan raw material (B) Cefotaxime raw material (C) bare CSNPs (D) cefotaxime loaded CSNPs.** FTIR spectrum of powders were taken after mixing raw material with KBr. Whereas liquid samples were analyzed directly by palcing a tiny drop over glass assembly. FTIR spectra indicated no new bond formation so the drug is not reacting chemically with nano-scaffolds.

Fourier Transform Infrared Spectroscopy analysis of chitosan raw material, cefotaxime powder empty CSNPs and drug loaded CSNPs are shown in **Figure 4A**. FTIR spectra of CS raw material revealed major peaks at 3401, 2878, 1651, 1380, 1080, and at 607 cm^-1^ that corresponded to O-H stretching peak, C-H stretching, N-H stretch, CH_2_ bending, C-O-C stretch and -C ≡ C-H, respectively (**Figure 4A**).

In case of CSNPs more broad, strong and characteristic peak for CS was observed at 3406 cm^-1^ representing 0-H stretching or H-bonding for alcohol or phenols instead of 3401 cm^-1^ (for CS) indicating that H bonding is enhanced during NPs formation while the amide peak shifted from 1651 to 1644 cm^-1^. A new sorption bands at 2089 cm^-1^ appear, which shows that the ammonium groups are crosslinked with tripolyphosphate molecules.

Analysis of FTIR spectra of CSNPs showed that it was very similar to that of cefotaxime loaded CSNPs (**Figures 4C,D**). This fact was predictable because the amount of drug loaded in nano-scaffold was very small as compare to the amount of building blocks of nano-structures, that is why no peaks of cefotaxime was observed in the drug loaded nanoparticles.

### Determination of Zeta Potential

**FIGURE 5:**
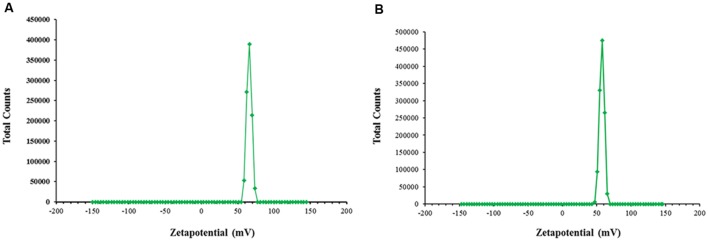
**Zeta potential.** Measured by Malvern Zeta sizer at ambient conditions **(A)** empty CSNPs **(B)** β-lactam drug loaded CSNPs. Both empty and drug loaded CSNPs are displaying zeta potential values greater than 50 mV. It indicates highly stable colloidal dispersion.

According to our results, both the blank CSNPs and drug loaded CSNPs had demonstrated a zeta potential of more than +50 mV (**Figures 5A,B**). This can be predicted from the data that β-lactam loaded nano-sytems are offering long term stability.

### Determination of Encapsulation Efficiency

Encapsulation efficiency was determined at different drug concentrations. It was observed that EE augmented with increase in concentration of drug. It was calculated to be 60% at 100 μg/mL; 71% at 800 μg/mL and 90% at 2000 μg/mL concentration.

### Antimicrobial Potential of the Prepared Nano-antibiotics

**FIGURE 6:**
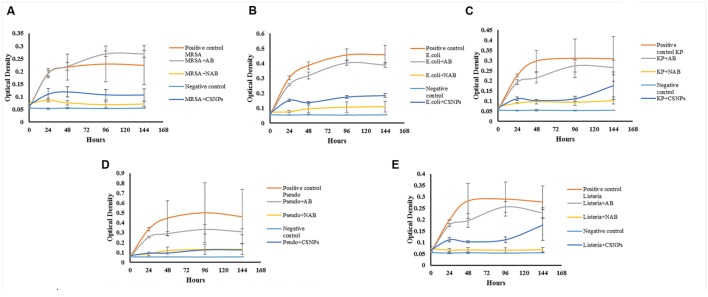
**Comparative Anti-pathogenic ability of Aqueous solution of Antibiotic and Antibiotic loaded CSNPs against pathogens (A) Methicillin-resistant *Staphylococcus aureus* (MRSA), (B) *Escherichia coli* (*E. coli*) (C), *Klebsiella pneumoniae* (KP), (D) *Pseudomonas aeruginosa (Pseudo)* and (E) *Listeria monocytogenes (Listeria).*** Pathogens were exposed to Antibiotic solution (AB), blank chitosan nano-particles (CSNPs), and antibiotic loaded CSNPs (NAB). All samples were incubated at 37°C for 144 h and after every 24 h reading were taken on ELISA plate reader at 595 nm. Negative control contains only the media and no inoculum where as positive control contain inoculated media.

**FIGURE 7:**
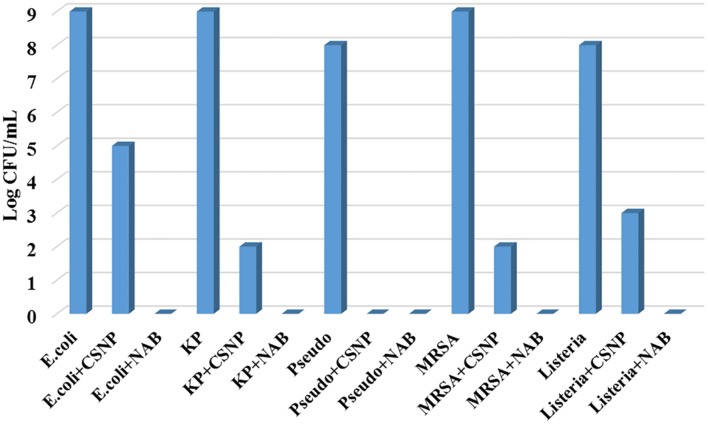
**Colony forming unit (CFU) assay.** CFU assay was performed to count the viable bacteria after reaction of CSNPs and drug loaded CSNPs with pathogens. CFU was done by plating 10μL of sample from each test tube after serial dilutions in normal saline (0.9% NaCl). All plates were then incubated for 48 h and colonies were counted manually. CSNP, Chitosan nano-particles, NAB, Nano-antibiotic; KP, *Klebsiella pneumoniae*; *Pseudo*, *Pseudomonas aeruginosa* and MRSA for Methicillin-resistant *S. aureus.*

Drug loaded CSNPs were prepared with 1000 μg/mL of drug concentration and tested against above-mentioned pathogens by broth dilution assay (**Figure 6**). It was compared to both plain antibiotic solution and bare CSNPs for 7 days (144 h). It was observed that nano-cefotaxime was highly effective against all MDR pathogens while the simple antibiotic could not control them at all. However, when we compare efficiency of drug loaded CSNPs to bare CSNPs, the difference seems to be low. In case of *P. aeruginosa* the activity was equal. However, for other pathogens bare nano-systems were effective till first 48 h. Nonetheless, the drug loaded CSNPs were proved to be more effective in contending the other four pathogens including ATCC *L. monocytogenes* for more prolonged period of time. At the end of experiment CFU count was done to confirm the hypothesis (**Figure 7**). It also endorse the above statement as no colonies were observed in case of *P. aeruginosa*, however, in other pathogens growth was observed only in case of CSNPs.

### Anti-Biofilm Activity of the Fabricated Nano-antibiotics

**FIGURE 8:**
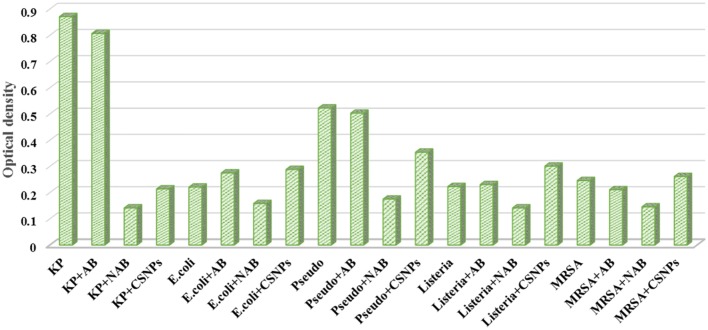
**Anti-biofilm activity of antibiotic suspension (AB) Chitosan nano-particles (CSNPs) and Cefotaxime loaded CSNPs (NAB) against pathogens.** Methicillin-resistant *S. aureus* (MRSA), *Klebsiella pneumoniae* (KP), *Escherichia coli (E. coli), Pseudomonas aeruginosa (Pseudo)*, and *Listeria monocytogenes (Listeria)* were incubated on ELISA plates at 37°C for 24 h. Biofilms were stained by crystal violet stain and removed by 30% acetic acid solution. This solution was then measured on ELISA plate reader at 595 nm.

It was observed from the present study that the formation and intensity of biofilm increase with the intensity of pathogenicity and resistance spectrum of pathogens. The most resistant Gram-negative pathogens were *K. pneumoniae* and *P. aeruginosa* and they formed more intense biofilms. Whereas, *L. monocytogenes* (ATCC) was most susceptible to antibiotic disks and was the weakest in term of biofilm intensity. Antibiotic alone fails to eradicate the biofilm, however, nano-cefotaxime was found to be highly effective in controlling the formation of biofilms (**Figure 8**). CSNPs were also found to be efficient in controlling the biofilms but the drug loaded CSNPs were more effective in every case. It may be concluded that bare CSNPs can effectively kill the planktonic cells, however, cannot efficiently control biofilm formation. Whereas drug loaded CSNPs were more effective as a therapeutic agent to prevent the formation of biofilm and also in terms of killing pathogens for prolonged period of time.

## Discussion

Biofilms are defined as microbial communities of cells that are irreversibly attached to a substratum or to an interface or to each other, and are embedded into a matrix of EPSs that they have produced. There are as many different types of biofilms as are bacteria, and that a single bacterium may even make several different types of biofilms under different environmental conditions (Karatan and Watnick, 2009). These biofilms are offering inherent barrier to the penetration of conventional antibiotics. Though anti-biofilm potential of plant extracts was reported by many others (Perumal and Mahmud, 2013), yet this study has been designed to sort out the potential of antibiotic loaded chitosan NPs as an antibacterial and anti-biofilm agents. Cefotaxime has been used for this purpose. Albeit, numerous methods are available for qualitative and quantitative assays of antibiotics, nevertheless the UV-Vis Spectrophotometric method has proved to be simple, effective, fast, economical and reproducible for quantification of cefotaxime sodium in pharmaceutical form powder for injectable solution. Resultantly, AAAA_max_ was obtained at 298 nm. Similar method was also employed by other authors though different AAAA_max_ values were achieved by them. According to Bushra et al. (2014) AAAA_max_ value for cefotaxime was obtained at 260 nm, at 228 nm (El-Shaboury et al., 2007), and 253 nm (Hammood et al., 2011). NPs were formulated by ionic gelation method that has proved to be most efficient and convenient method. Engineered and naturally forming NPs can vary widely in their physicochemical characteristics such as shape, size, and charge. These characteristics have been reported to impact their interactions with biofilm-coated surfaces (Ikuma et al., 2015). Microscopic attributes were studied via AFM and SEM wherein the results displayed much smaller size of NPs as compared to previous studies. Previous studies have reported the size of CSNPs in the range of 140–250 nm (Gan et al., 2005; Nesalin and Smith, 2013; Chaubey and Mishra, 2014; Miladi et al., 2015) while in this research size less than 100 nm was achieved.

Fourier Transform Infrared Spectroscopy spectroscopic studies were carried out in order to confirm the formation of NPs and type of interactions between antibiotic and polymer. FTIR has proved to be a powerful and useful characterization method for polymers, and materials in general. This is quite an economical, short time characterization that allows to establish the chemical composition, microstructure, chemical interactions and follow variation of specific functional groups with the passage of time during reactions (Barrios et al., 2012).

Fourier Transform Infrared Spectroscopy spectrum of chitosan showed resemblance with an already reported spectra for this biopolymer (Khan et al., 2002; Qi et al., 2004; Vitali et al., 2006). Spectrum of cefotaxime raw material was also in accordance with previously reported spectrum for this cephalosporin drug (Hammood et al., 2011). Likewise, FTIR spectrum of empty CSNPs was similar to already reported spectra, however, it was quite different from the spectrum of bulk material that indicated the formation of NPs (Hosseini et al., 2013; de Pinho Neves et al., 2014; Antoniou et al., 2015). The spectrum for drug loaded NPs was almost superimposable to that of empty nano-formlation indicating that there was no chemical bonding between the drug and polymers (**Figures 4C** and **3B**).

Zeta potential is an indicator of long term stability of colloidal dispersions. Nanoparticles with a zeta potential between -10 and +10 mV are considered approximately neutral, while nanoparticles with zeta potentials of greater than +30 mV or less than -30 mV are considered strongly cationic and strongly anionic, respectively (Clogston and Patri, 2011) and represent a stable formulation as there would be less aggregation and flocculation as the repulsive forces become more strong (Gazori et al., 2009). According to results, both the blank CSNPs and drug loaded CSNPs were displaying highly positive zeta potential of more than +50 mV (**Figures 5A,B**). This can be predicted from the data that β-lactam loaded nano-systems are offering long term stability. Positive zeta potential in the range of 21–45 was already reported by many authors (De Campos et al., 2001; Miladi et al., 2015; Zhang et al., 2016).

Encapsulation efficiency was determined at various concentrations of cefotaxime to check the influence of drug concentration on EE. It was observed that EE augmented with increase in concentration of drug. However, the findings indicated that if the drug was dissolved in CS solution and TPP was added afterward it had resulted in very low EE (<3%). Honary et al. (2014) encapsulated vancomycin in CSNPs and reported 60–69% EE (Honary et al., 2014) while 42–55% after encapsulation of 5-Fluorouracil. The well –characterized CSNPs were further investigated for their antipathogenic and anti-biofilm potential.

Bacterial biofilms were first described in 1943. Biofilm formation is an intricate process and colloquially the biofilms are also referred to as microbial cities. Preliminary step to this phenomenon is the attachment to any solid surface termed as ‘adsorption.’ These biofilms are sheltered by a self-secretory matrix called EPS. This EPS hold the cells organized and safeguard them against extraneous agents because of its composition. Biofilms also aid in quorum sensing thus facilitating microbes to communicate with each other. The composition of EPS is protein, polysaccharides and extracellular DNA. Biofilms can ensure maximum intake of nutrients from outside environment. The organization of microbes in these biofilms is such that ancient and dead cell recline in the center where the metabolites get accumulated while the fresh and new cells lie near the outer surface where the flow of materials is maximum. These biofilms are offering an innate resistance mechanism to conventional antibiotics, however, NAMs can encounter biofilms successfully.

The interactions between NPs and the biofilm can be viewed as a three-step process: (1) transport of NPs to the vicinity of the biofilm; (2) attachment to the biofilm surface; and (3) migration within the biofilms (Ikuma et al., 2015).

It has been observed from the present study that the nano-cefotaxime was highly effective in controlling the formation of biofilms (**Figure 8**). Though anti-biofilm potential of plant extracts has been reported by many others (Perumal and Mahmud, 2013), however, this is the first report to prove the anti-biofilm activity of cefotaxime loaded CSNPs. It has been mentioned earlier that more than 70% pathogenic microbes are biofilm forming and that is the main mechanism of resistance in pathogens. Blank CSNPs also exhibited good potential in lowering the biofilm formation intensity but fails to eradicate it completely. Therefore it can be concluded that chitosan NPs causes the clumping of bacterial mass and help to slow the growth of pathogens, however, cannot control the biofilm formation efficiently. Less intensity of biofilm is the direct result of less number of pathogens in culture media. The drug loaded CSNPs totally eradicated the pathogens and therefore biofilm formation.

## Conclusion

Foregoing in view, it can be positively concluded that the main mechanism of action by which NAMs control MDR pathogens is inhibition of biofilm formation. The results in this research have confirmed that chitosan can be a potential carrier system for cefotaxime to target Gram-negative and Gram-positive multi drug resistant microorganisms that have the tendency to form microbial biofilms. The prepared NPs have been perceived to be stable as carrying positive zeta of more than +50 mV and were displaying homogeneity in both size and shape.

## Author Contributions

BJ designed, developed, and performed all the experimental work, analysed and interpreted the data and wrote the manuscript. HH performed and helped in analyzing the Atomic Force Microscopy (AFM) Assays. SA helped in collection of clinical isolates and their characterization. AI provided technical assistance for Zeta sizer analysis. HN done the FTIR analysis and its interpretation. MI supervised the work at each step, provided the budget and scientific assistance for the manuscript write-up.

## Conflict of Interest Statement

The authors declare that the research was conducted in the absence of any commercial or financial relationships that could be construed as a potential conflict of interest.

## References

[B1] Allaker R. P. Memarzadeh K. (2014). Nanoparticles and the control of oral infections. *Int. J. Antimicrob. Agents* 43 95–104. 10.1016/j.ijantimicag.2013.11.002 24388116

[B2] Antoniou J. Liu F. Majeed H. Qi J. Yokoyama W. Zhong F. (2015). Physicochemical and morphological properties of size-controlled chitosan–tripolyphosphate nanoparticles. *Colloids Surf. A Physicochem. Eng. Asp.* 465 137–146. 10.1016/j.colsurfa.2014.10.040

[B3] Barrios V. A. E. Espinosa G. A. Rodríguez J. L. D. Méndez J. R. R. Aguilar N. V. P. (2012). *FTIR-An Essential Characterization Technique for Polymeric Materials.* Rijeka: InTech Open Access Publisher.

[B4] Bushra M. U. Akter N. Hassan M. R. Islam A. Hossain M. R. (2014). Development and validation of a simple UV spectrophotometric method for the determination of cefotaxime sodium in bulk and pharmaceutical formulation. *IOSR J. Pharm.* 4 74–77.

[B5] Carlet J. Jarlier V. Harbarth S. Voss A. Goossens H. Pittet D. (2012). Ready for a world without antibiotics? The pensières antibiotic resistance call to action. *Antimicrob. Resist. Infect. Control* 1 1–13. 22958833 10.1186/2047-2994-1-11 PMC3436635

[B6] Chaubey P. Mishra B. (2014). Mannose-conjugated chitosan nanoparticles loaded with rifampicin for the treatment of visceral leishmaniasis. *Carbohydr. Polym.* 101 1101–1108. 10.1016/j.carbpol.2013.10.044 24299880

[B7] Clogston J. D. Patri A. K. (2011). “Zeta potential measurement,” in *Characterization of Nanoparticles Intended for Drug Delivery*, ed. McNeil S. E. (New York, NY: Humana Press), 63–70.

[B8] De Campos A. M. Sánchez A. Alonso M. J. (2001). Chitosan nanoparticles: a new vehicle for the improvement of the delivery of drugs to the ocular surface. Application to cyclosporin A. *Int. J. Pharm.* 224 159–168. 10.1016/S0378-5173(01)00760-8 11472825

[B9] de Pinho Neves A. L. Milioli C. C. Müller L. Riella H. G. Kuhnen N. C. Stulzer H. K. (2014). Factorial design as tool in chitosan nanoparticles development by ionic gelation technique. *Colloids Surf. A Physicochem. Eng. Asp.* 445 34–39. 10.1016/j.colsurfa.2013.12.058

[B10] Delcour A. H. (2009). Outer membrane permeability and antibiotic resistance. *Biochim. Biophys. Acta* 1794 808–816. 10.1016/j.bbapap.2008.11.005 19100346 PMC2696358

[B11] Dheilly A. Soum-Soutéra E. Klein G. L. Bazire A. Compère C. Haras D. (2010). Antibiofilm activity of the marine bacterium *Pseudoalteromonas* sp. strain 3J6. *Appl. Environ. Microbiol.* 76 3452–3461. 10.1128/AEM.02632-09 20363799 PMC2876442

[B12] Donlan R. M. (2002). Biofilms: microbial life on surfaces. *Emerg. Infect. Dis.* 8 881–890. 10.3201/eid0809.020063 12194761 PMC2732559

[B13] Du W. L. Niu S. S. Xu Y. L. Xu Z. R. Fan C. L. (2009). Antibacterial activity of chitosan tripolyphosphate nanoparticles loaded with various metal ions. *Carbohydr. Polym.* 75 385–389. 10.1016/j.carbpol.2008.07.039

[B14] El-Shaboury S. R. Saleh G. A. Mohamed F. A. Rageh A. H. (2007). Analysis of cephalosporin antibiotics. *J. Pharm. Biomed. Anal.* 45 1–19. 10.1016/j.jpba.2007.06.002 17689910

[B15] Fabbretti A. Gualerzi C. O. Brandi L. (2011). How to cope with the quest for new antibiotics. *FEBS Lett.* 585 1673–1681. 10.1016/j.febslet.2011.04.029 21513713

[B16] Gan Q. Wang T. Cochrane C. McCarron P. (2005). Modulation of surface charge, particle size and morphological properties of chitosan–TPP nanoparticles intended for gene delivery. *Colloids Surf. B Biointerfaces* 44 65–73. 10.1016/j.colsurfb.2005.06.001 16024239

[B17] Gazori T. Khoshayand M. R. Azizi E. Yazdizade P. Nomani A. Haririan I. (2009). Evaluation of Alginate/Chitosan nanoparticles as antisense delivery vector: formulation, optimization and in vitro characterization. *Carbohydr. Polym.* 77 599–606. 10.1016/j.carbpol.2009.02.019

[B18] George A. (2011). Microtiter dish biofilm formation assay. *J. Vis. Exp.* 47 2437 10.3791/2437 PMC3182663 21307833

[B19] Hajipour M. J. Fromm K. M. Ashkarran A. A. Jimenez de Aberasturi D. de Larramendi I. R. Rojo T. (2012). Antibacterial properties of nanoparticles. *Trends Biotechnol.* 30 499–511. 10.1016/j.tibtech.2012.06.004 22884769

[B20] Hammood M. K. Qasim A. W. Jasim F. (2011). An indirect atomic absorption spectrophotometric determination of cefotaxime in pharmaceutical formulations by using rhodium (II) as a mediating metal. *Nat. J. Chem.* 41 27–37.

[B21] Hetrick E. M. Shin J. H. Paul H. S. Schoenfisch M. H. (2009). Anti-biofilm efficacy of nitric oxide-releasing silica nanoparticles. *Biomaterials* 30 2782–2789. 10.1016/j.biomaterials.2009.01.052 19233464 PMC2692680

[B22] Honary S. Ebrahimi P. Hadianamrei R. (2014). Optimization of particle size and encapsulation efficiency of vancomycin nanoparticles by response surface methodology. *Pharm. Dev. Technol.* 19 987–998. 10.3109/10837450.2013.846375 24147898

[B23] Hosseini S. F. Zandi M. Rezaei M. Farahmandghavi F. (2013). Two-step method for encapsulation of oregano essential oil in chitosan nanoparticles: preparation, characterization and in vitro release study. *Carbohydr. Polym.* 95 50–56. 10.1016/j.carbpol.2013.02.031 23618238

[B24] Ikuma K. Decho A. W. Lau B. L. (2015). When nanoparticles meet biofilms-Interactions guiding the environmental fate and accumulation of nanoparticles. *Front. Microbiol.* 6:591 10.3389/fmicb.2015.00591 PMC4468922 26136732

[B25] Jamil B. Bokhari H. Imran M. (2015). Mechanism of action: how nano-antimicrobials act? *Curr. Drug Targets* 10.2174/1389450116666151019101826 [Epub ahead of print]. 26477460

[B26] Jamil B. Habib H. Abbasi S. Nasir H. Rahman A. Rehman A. (2016). Cefazolin loaded chitosan nanoparticles to cure multi drug resistant Gram-negative pathogens. *Carbohydr. Polym.* 136 682–691. 10.1016/j.carbpol.2015.09.078 26572401

[B27] Karatan E. Watnick P. (2009). Signals, regulatory networks, and materials that build and break bacterial biofilms. *Microbiol. Mol. Biol. Rev.* 73 310–347. 10.1128/MMBR.00041-08 19487730 PMC2698413

[B28] Khan T. A. Peh K. K. Chang H. S. (2002). Reporting degree of deacetylation values of chitosan: the influence of analytical methods. *J. Pharm. Pharm. Sci.* 5 205–212. 12553887

[B29] Kirker K. R. Fisher S. T. James G. A. (2015). Potency and penetration of telavancin in staphylococcal biofilms. *Int. J. Antimicrob. Agents* 46 451–455. 10.1016/j.ijantimicag.2015.05.022 26213381

[B30] Lewis K. (2005). Persister cells and the riddle of biofilm survival. *Biochemistry (Mosc.)* 70 267–274. 10.1007/s10541-005-0111-6 15807669

[B31] Madsen J. S. Burmølle M. Hansen L. H. Sørensen S. J. (2012). The interconnection between biofilm formation and horizontal gene transfer. *FEMS Immunol. Med. Microbiol.* 65 183–195. 10.1111/j.1574-695X.2012.00960.x 22444301

[B32] Miladi K. Sfar S. Fessi H. Elaissari A. (2015). Enhancement of alendronate encapsulation in chitosan nanoparticles. *J. Drug Deliv. Sci. Technol.* 30 391–396. 10.1016/j.jddst.2015.04.007

[B33] Nesalin J. A. J. Smith A. A. (2013). Preparation and evaluation of stavudine loaded chitosan nanoparticles. *J. Pharm. Res.* 6 268–274. 10.1016/j.jopr.2013.02.004

[B34] Patil P. Chavanke D. Wagh M. A. (2012). Review on ionotropic gelation method: novel approach for controlled gastroretentive gelispheres. *Int. J. Pharm. Pharm. Sci.* 4 27–32.

[B35] Pelgrift R. Y. Friedman A. J. (2013). Nanotechnology as a therapeutic tool to combat microbial resistance. *Adv. Drug Deliv. Rev.* 65 1803–1815. 10.1016/j.addr.2013.07.011 23892192

[B36] Perumal S. Mahmud R. (2013). Chemical analysis, inhibition of biofilm formation and biofilm eradication potential of *Euphorbia hirta* L. against clinical isolates and standard strains. *BMC Complement. Altern. Med.* 13:346 10.1186/1472-6882-13-346 PMC4029191 24321370

[B37] Qi L. Xu Z. Jiang X. Hu C. Zou X. (2004). Preparation and antibacterial activity of chitosan nanoparticles. *Carbohyd. Res.* 339 2693–2700. 10.1016/j.carres.2004.09.007 15519328

[B38] Rotar O. V. Tenedja K. Arkhelyuk A. D. Rotar V. I. Davidencko I. S. Fediv V. I. (2014). Preparation of chitosan nanoparticles loaded with glutathione for diminishing tissue ischemia-reperfusion injury. *Int. J. Adv. Eng. Nano Technol.* 1 19–23.

[B39] Salta M. Wharton J. A. Dennington S. P. Stoodley P. Stokes K. R. (2013). Anti-biofilm performance of three natural products against initial bacterial attachment. *Int. J. Mol. Sci.* 14 21757–21780. 10.3390/ijms141121757 24192819 PMC3856033

[B40] Sayem S. M. Manzo E. Ciavatta L. Tramice A. Cordone A. Zanfardino A. (2011). Anti-biofilm activity of an exopolysaccharide from a sponge-associated strain of *Bacillus licheniformis*. *Microb. Cell Fact.* 10 74 10.1186/1475-2859-10-74 PMC3196911 21951859

[B41] Smith A. W. (2005). Biofilms and antibiotic therapy: is there a role for combating bacterial resistance by the use of novel drug delivery systems? *Adv. Drug Deliv. Rev.* 57 1539–1550. 10.1016/j.addr.2005.04.007 15950314

[B42] Stewart P. S. (2002). Mechanisms of antibiotic resistance in bacterial biofilms. *Int. J. Med. Microbiol.* 292 107–113. 10.1078/1438-4221-00196 12195733

[B43] Suresh A. K. (2015). “Analytical and physical characterization techniques employed to assess microbial toxicity of nanoparticles,” in *Co-Relating Metallic Nanoparticle Characteristics and Bacterial Toxicity*, ed. Suresh A. K. (New York, NY: Springer International Publishing), 15–26.

[B44] Vitali L. Justi K. C. Laranjeira M. Fávere V. T. (2006). Impregnation of chelating agent 3, 3-bis-N, N bis-(carboxymethyl) aminomethyl-o-cresolsulfonephthalein in biopolymer chitosan: adsorption equilibrium of Cu (II) in aqueous medium. *Polímeros* 16 16–122. 10.1590/S0104-14282006000200011

[B45] Watnick P. Kolter R. (2000). Biofilm, city of microbes. *J. Bacteriol.* 182 2675–2679. 10.1128/JB.182.10.2675-2679.2000 10781532 PMC101960

[B46] Zhang H. Jung J. Zhao Y. (2016). Preparation, characterization and evaluation of antibacterial activity of catechins and catechins-Zn complex loaded β-chitosan nanoparticles of different particle sizes. *Carbohydr. Polym.* 137 82–91. 10.1016/j.carbpol.2015.10.036 26686108

